# Prior Compensation Algorithm for Cerenkov Luminescence Tomography From Single-View Measurements

**DOI:** 10.3389/fonc.2021.749889

**Published:** 2021-09-23

**Authors:** Lin Wang, Xiaowei He, Jingjing Yu

**Affiliations:** ^1^ School of Information Sciences and Technology, Northwest University, Xi’an, China; ^2^ School of Computer Science and Engineering, Xi’an University of Technology, Xi’an, China; ^3^ School of Physics and Information Technology, Shaanxi Normal University, Xi’an, China

**Keywords:** Cerenkov luminescence tomography (CLT), prior compensation, optical imaging (OI), cancer, tomographic reconstruction

## Abstract

Cerenkov luminescence tomography (CLT) has attracted much attention because of the wide clinically-used probes and three-dimensional (3D) quantification ability. However, due to the serious morbidity of 3D optical imaging, the reconstructed images of CLT are not appreciable, especially when single-view measurements are used. Single-view CLT improves the efficiency of data acquisition. It is much consistent with the actual imaging environment of using commercial imaging system, but bringing the problem that the reconstructed results will be closer to the animal surface on the side where the single-view image is collected. To avoid this problem to the greatest extent possible, we proposed a prior compensation algorithm for CLT reconstruction based on depth calibration strategy. This method takes full account of the fact that the attenuation of light in the tissue will depend heavily on the depth of the light source as well as the distance between the light source and the detection plane. Based on this consideration, a depth calibration matrix was designed to calibrate the attenuation between the surface light flux and the density of the internal light source. The feature of the algorithm was that the depth calibration matrix directly acts on the system matrix of CLT reconstruction, rather than modifying the regularization penalty items. The validity and effectiveness of the proposed algorithm were evaluated with a numerical simulation and a mouse-based experiment, whose results illustrated that it located the radiation sources accurately by using single-view measurements.

## Introduction

Cerenkov luminescence imaging (CLI) provides a potential solution to the problem of clinical translation of optical imaging, because many radionuclide probes that are widely used in clinic can be used as the light emission source of CLI. Since Robertson used a CCD camera to collect the Cerenkov luminescence (CL) from a small animal in 2009, the CLI has been rapidly developed and widely used in the biomedical fields (1–5). However, CLI is a method of two-dimensional (2D) planar imaging, which cannot provide the three-dimensional (3D) spatial distribution of the radionuclide probes. Thus, it cannot accurately quantify and analyze these probes as well as the molecules they target (6). This problem can be solved by its 3D derivatives, namely the Cerenkov luminescence tomography (CLT). CLT reconstructs the spatial distribution of the internal radionuclide probes by integrating the CL images measured from the body surface with the structural information and other prior information (7–13). Li et al. first proposed the concept of CLT, reconstructing the 3D distribution of ^18^FDG in a homogeneous mouse model (8). The homogenous model is relatively simple, and is quite different from the real imaging organisms, resulting in inaccurate results. This can be solved by more complex heterogeneous model (8, 9, 11, 14, 15). However, due to the serious morbidity of CLT reconstruction, the quality of the reconstructed images needs to be further improved. There are several ways to reduce the ill-posedness and improve the quality of the reconstruction results, including combining the strategies of permissible source region, multi-view or multispectral measurements, and regularization terms (16–19). These strategies are designed to improve the ill-posedness by reducing the dimension of the domain to be solved, increasing the dimension and number of measurements, or regulating regularization penalties. Increasing the amount of measured data is one of commonly used methods. However, a large amount of reconstruction data undoubtedly increases the reconstruction time and limit the reconstruction efficiency. Using a small amount of data to obtain accurate reconstruction results is an important problem to be solved at this stage.

In the process of image acquisition of CLI, especially when using a commercial system, small animals usually lie flat on the animal holder. In this case, only the CL light emitted from the top surface of the animal can be collected. This leads to the need of 3D reconstruction based on incomplete or single-view images. Single-view based reconstruction can reduce data acquisition time, and can use less data to reconstruct the targeted probes within live organisms (20). However, the reconstructed results based on the single-view image will be closer to the animal surface on the side where the single-view image is collected. For single-view imaging, the light from the point near the surface of the imaging object contributes more to the collected image, whereas the point far away contributes less. This is due to the lack of other views signals, resulting in the detector’s detection sensitivity to points far away from the detection surface becomes weak. Liu proposed a depth compensation algorithm for diffuse optical tomography (DOT) (21–23), which compensates the detection sensitivity loss by constructing depth compensation matrix. However, in the DOT imaging mode, the light source is known and located outside the organism, which is different from the CLT with unknown light source located inside the organism.

Inspired by the depth compensation algorithm in DOT, and considering that the attenuation of light in tissues depends heavily on the depth of light emission source as well as the distance between the light emission source and the detection plane, here we proposed a prior compensation algorithm for CLT reconstruction based on depth calibration strategy. CL image detected from single-view results in the loss of other views of luminescent signals. The farther away from the detection plane, the greater the loss of the luminescent signals can be. Thus, a depth calibration matrix was designed to calibrate the signal loss between the detected surface light flux and the density of internal light source. The depth calibration matrix has the same dimensions as CLT system matrix, and is composed of different compensation weights. Here, the compensation weights are determined using the detection plane as a benchmark, which is where they differ from those in existing studies (21–23). In the calibration process, the larger weight will be given to the points far away from the detection plane, and the smaller weight will be given to the points near. This calibration can balance the detection sensitivity loss in depth direction due to the increase of depth. With the help of this strategy, the inaccuracy of CLT reconstruction from single-view image can be improved. We evaluate the validity and effectiveness of the proposed method with a numerical simulation and a mouse-based experiment.

## Methods

When the fluctuation of CL is neglected, the radiative transfer equation (RTE) can be used to accurately describe the propagation process of CL light in medium. However, in the practical application, it is hard to imagine the calculation cost of solving RTE directly. Several compromised schemes for RTE have been proposed, such as Monte Carlo computational model based on statistics and the approximate models based on numerical simplification. Among these approximate models, the diffusion equation (DE), simplified spherical harmonics approximation (SP_N_), and their hybrid equation are usually used in 3D optical imaging (24–26). Due to the high accuracy and high computational efficiency in the high diffusion medium, DE has been widely used in the 3D optical imaging of living animals. The expression of the DE is:


(1) $$\{\begin{array}{ll} -\nabla \cdot \left(D\left(r\right)\nabla \phi \left(r\right)\right)+\mu_{a}\left(r\right)\phi \left(r\right)=Q\left(r\right) & r\in \Omega \\ J\left(r\right)=-D\left(r\right)\left(v\cdot \nabla \phi \left(r\right)\right) & r\in \partial \Omega \end{array}$$

where Ω is the region of interest to be solved, and ∂Ω is its boundary; *ø* (*r*) is the nodal flux density at position *r*;*Q (r)* denotes the power density of the radionuclide probes; *D (r)* is the diffusion coefficient that is related to the absorption coefficient and the reduced scattering coefficient; *μ_a_ (r)* represents the absorption coefficient; *v* is the unit outer normal on ∂Ω; and *J (r)* is the outgoing light flux density measured on the outer boundary.

Through a series of operations, including transforming Eq (1). into weak form, the tetrahedral mesh discretization, and the finite element calculation, Eq (1). can be converted to the following linear equation (27):


(2) MΦ=FQ

where *Q* denotes the source matrix, and *F* is the source weighted matrix; Φ is a nodal flux density related matrix; and *M* is a sparse positive definite stiffness matrix describing the relationship between the power density of the light source and the measurable nodal flux density. This relationship is determined by the absorption and scattering of tissue, and is highly dependent on the distance between two nodes.

To calibrate the effect of depth on the detection signal, a depth calibration matrix C was designed in this study. The depth calibration matrix has the same dimensions as CLT stiffness matrix, and is composed of different compensation weights. Different from the existing studies (21–23), the compensation weights used in C were determined using the detection plane as a benchmark. In the calibration process, the larger weights will be given to the points far away from the detection plane, and the smaller weights will be given to the points near. After integrating the depth calibration matrix, the matrix equation of Eq (2). can be rewritten as:


(3) (MC)Φ=FQ

Here, the depth calibration matrix *C* is defined as:


(4) $$C={\left[Arr(C^{\#})\right]}^{\lambda }$$

where γ is an adjustable parameter between 0 and 3, determined empirically for practical applications; *Arr* (•) denotes an operation that rearranges all the elements of a matrix in an order like the original nodes in the *M* matrix; and *C^#^* is a diagonal matrix with the same dimension as *M*, and each element is a weighted value determined by the distance between the observed node and the detection plane. This matrix *C^#^* can be determined as follows. Firstly, the matrix *M* having a dimension of *m*×*m* was divided into *N* submatrix having a dimension of *m*/*N*×*m*. Secondly, the maximum eigenvalue of each submatrix *N_k_* (*k*=1, 2, …, *N*) was calculated, and the maximum eigenvalue of the *k*th submatrix was defined as *S_max,k_*. Thirdly, the average distance of the nodes in each submatrix to the detection plane was calculated, and the submatrices were then renumbered according to these distance values. The submatrix at the *t*th distance from the detection plane was noted as *N*_max,_ *_t_* (*t*=1, 2, …, *N*). For example, the farthest submatrix from the detection plane was assigned a new number as *N*_max,_ *_1_* and the nearest one was *N*_max,_ *_N_*. Finally, we sorted the *N* maximum eigenvalues from largest to smallest, and assigned the larger values to the positions of the submatrices with smaller *t* values. These sorted eigenvalues formed the *C^#^* matrix as the values of the elements on its diagonal. For example, the largest value was assigned to the position in the *C^#^* matrix where the submatrix *N_K_* furthest from the detection plane was located.

Eq (3). can be further converted into the following linear matrix equation between the internal radionuclide source and the boundary measurement:


(5) J=AQ

where *J* is the exiting light flux current measured at boundary; and $A={\left[{\left(MC\right)}^{–1}F\right]}_{\Theta }$ with ${\left[•\right]}_{\Theta }$ representing an operation of removing the elements in the specified matrix that corresponds to the light flux density at the internal nodes.

Equation (6) is the system matrix of the proposed algorithm for single-view CLT. Since the radionuclide probes are sparse distributed in the animal body and the single-view measurement is seriously insufficient, the solution to Eq (6). can be converted into solving the following sparse regularization problem:


(6) $$Q=\arg\min\frac{1}{2}{\left||AQ-J|\right|}_{2}^{2}+\mu {\left|Q\right|}_{1}$$

where *μ* is the regularization parameter, and can be determined empirically in the experiments. In this work, the primal-dual interior-point algorithm was selected to solve Eq (7) (28). In the reconstruction, the initial position of source node was guessed at the center of the solving domain, which was used to construct the depth calibration matrix C.

## Experiments and Results

Two groups of experiments, including a numerical simulation and a mouse-based *in vivo* experiment were conducted to validate the performance of the proposed method. Two indicators were selected to quantitatively evaluate the reconstructed results. The first indicator is the distance error (Dis_Err) that defines the distance between the central position of reconstructed source and the actual center of the radionuclide probe. The second indicator is the depth error (Dep_Err) which is defined as the depth of the reconstructed source minus the depth of the actual radionuclide probe. This depth is relative to the detection plane of the single-view image, that is, the distance from the source to the detection plane is defined as the depth. Moreover, in order to evaluate the superiority of the proposed method, the DE method without depth calibration is selected as a reference for comparison.

### Numerical Simulation

**Figure 1:**
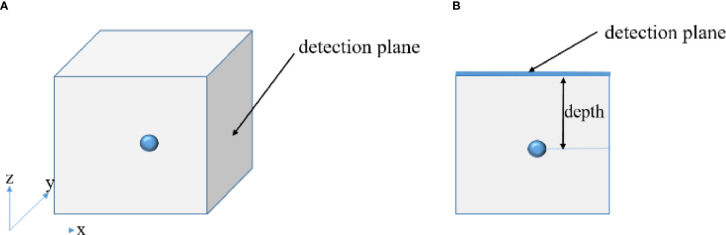
Schematic diagrams of the simulation model indicating where the single-view image was collected **(A)** and of the definition of the depth **(B)**.

In the numerical simulation, to show the advantages of the proposed method in depth reconstruction intuitively, a cube having a side length of 10 cm was selected as the simulation model. The absorption coefficient of the cube is 0.08 mm^-1^, and the reduced scattering coefficient is 10 mm^-1^. A sphere having a radius of 5 mm was employed as the light source, acting as the radionuclide probes. In order to investigate the performance of the depth compensation method, we placed the light source at different depths of 1 cm, 3 cm, and 5 cm from the detection surface respectively. **Figure 1** shows the diagram of the simulation model (**Figure 1** **A**) and the depth definition (**Figure 1B**). The measured image on the detected surface was obtained by solving the SP_3_ equation on the highly dense grid with the finite element method. The cube model was divided into 103,545 tetrahedrons and 18,471 nodes to form the highly dense grid. Single-view image from one of the planes of the cube was used as the measurements for reconstruction. In the process of the inverse reconstruction, the finite element discretization is completely different from that used in the forward simulation. The reconstruction was performed on a coarse mesh that consists of 18,262 tetrahedrons and 3,495 nodes. Finally, the proposed algorithm-based reconstruction method and the traditional DE method without combining the prior compensation algorithm were performed to locate the distribution of the mimic radioactive tracer.

**Figure 2:**
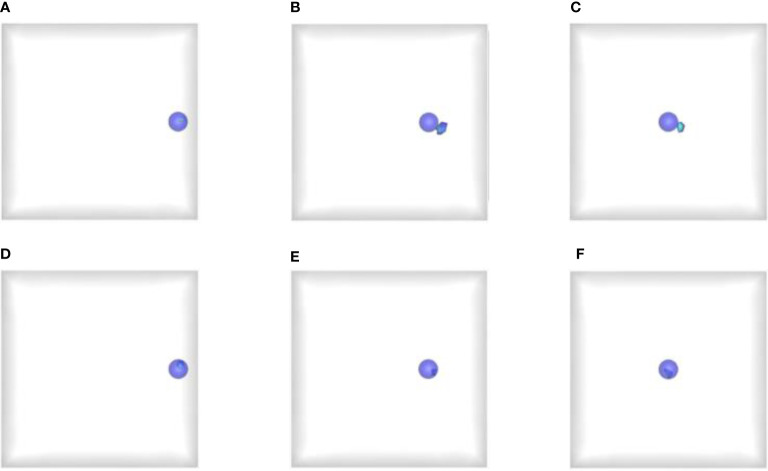
Reconstructed results obtained by the proposed and reference methods. **(A–C)** The reconstructed results of the reference method at depth of 1 cm, 3 cm and 5 cm respectively; **(D–F)** The reconstructed results of the proposed method at depth of 1 cm, 3 cm and 5 cm respectively. The blue solid spheres mark the actual distribution of the mimic radioactive tracer, and the colored tetrahedrons are the reconstructed source.

**Figure 2** shows the comparison results reconstructed by the proposed and reference methods. Therein, **Figures 2A** **–C** show the 3D reconstruction results of the reference method with the light source at depth of 1 cm, 3 cm and 5 cm respectively, and **Figures 2D–F** present the corresponding results of the prior compensation algorithm-based method. The blue solid spheres mark the actual distribution of the mimic radioactive tracer, and the colored tetrahedrons are the reconstructed source. First of all, we can find that when the depth of the light source was 1 cm, both the prior compensation algorithm-based method and the reference method can locate the distribution of the light source relatively accurately. This means that the attenuation of the signal transmission to the detection plane is not significant when the light source is located at shallow depths. However, as the depth of the light source increases, the attenuation of the signal transmission to the detection plane starts to become significant and can affect the results of the CLT reconstruction. For example, when the depth increased to 3 cm and 5 cm, the reference method cannot locate the light source well, and the reconstructed results tend to the surface on the side of the detection plane. Moreover, almost all the reconstructed tetrahedrons were outside the sphere where the actual light source was. On the contrary, the proposed algorithm can still locate the position of the light source accurately, and all the reconstructed tetrahedrons were concentrated inside the sphere where the light source was. These results confirmed that the *a priori* compensation algorithm is indeed effective for the reconstruction of deep light source and proved the advantages of the proposed method for the single-view reconstruction of deep light source.

**Figure 3:**
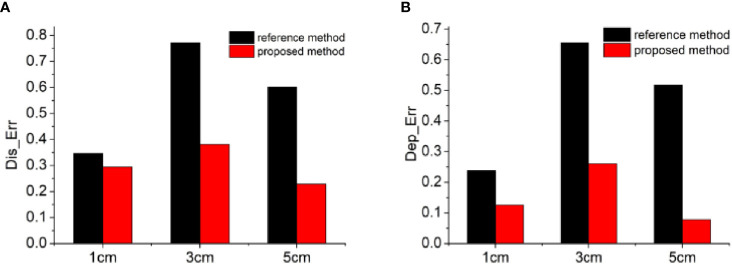
Quantitative analysis of the reconstruction results. The calculated values of the indicators of Dis_err **(A)** and Dep_err **(B)**. The black bars show the distance or depth errors of the reconstructed results by the reference method, and the red bars represent those obtained by the proposed method.

In order to quantitatively evaluate the reconstruction results, we calculated two indicators of Dis_Err and Dep_Err, as presented in **Figure 3**. Therein, the black bars show the distance or depth errors of the reconstructed results by the reference method, and the red bars represent those obtained by the proposed method. From these quantitative results, almost the same conclusion was addressed that the proposed method greatly improved the accuracy of the reconstruction results and reduced the depth positioning error, especially for the light sources at deeper depth. With the increase of the depth of the light source, the effect of this improvement became more obvious. For example, for the positioning accuracy of the light sources (**Figure 3** **A**), when the depth of the light source was 1 cm, the Dis_Err was similar for both methods, and the improvement ratio of the proposed method compared with the reference method was only 1.18. As the depth increased, the improvement ratio increased further, for example to 2.02 at 3 cm depth and 2.63 at 5 cm depth. For the depth resolving accuracy of the light sources (**Figure 3** **B**), a similar trend was obtained with the depth of the light source. As the depth increased from 1 cm to 5 cm, the improvement ratio of the proposed method compared with the reference one increased from 1.89 to 6.6. These quantitative results collectively demonstrated that the depth calibration matrix has a good regulatory effect on the depth of the reconstructed results, and the proposed algorithm can well compensate the inaccuracy of the reconstructed results caused by the data loss in the single-view image-based reconstruction.

### Mouse Based *In Vivo* Experiment

After the feasibility and effectiveness of the proposed method were verified with numerical simulation, we carried out a live mouse based *in vivo* experiment to further prove the application potential. In the experiment, an athymic male nude mouse was used as the imaging model, who is approximately six weeks old. All procedures were performed in accordance with the animal protocol of Xi’an Jiaotong University Animal Care and Use Committee, China (No. XJTULAC2016-412). Firstly, an artificial Cerenkov luminescent source was prepared, which was made of a glass vessel containing about 400 μCi ^18^F-FDG. The size of the artificial source was about 1 mm in diameter and 5 mm in length. The artificial source was then implanted into the liver of the living mouse. The mouse was placed on a flat animal platform after anesthesia, and put into the live small animal imaging system (IVIS Kinetic, PerkinElmer) for data collection. By using a filter, a luminescent image at 670 nm was collected on the top surface of the mouse for later reconstruction. The anatomical structural information of the mouse was obtained by our home-made rotating tube μCT system that is comprised of an X-ray tube (Series 5000, Oxford Instruments) and a flat panel detector (C7921CA-02, Hamamatsu). The anatomical structure contained some major organs, such as the lung, liver, heart, kidney, and muscle, their optical properties are listed in **Table 1** (29).

**Table 1:** Optical parameters of different organs in the mouse model.

Tissues	Muscle	Heart	Kidney	Lung	Liver
*μ_a_*	0.086201	0.058270	0.065341	0.194691	0.348867
*μ_s_*	0.429071	0.963871	2.253010	2.173884	0.678066

In units of mm^-1^.

**Figure 4:**
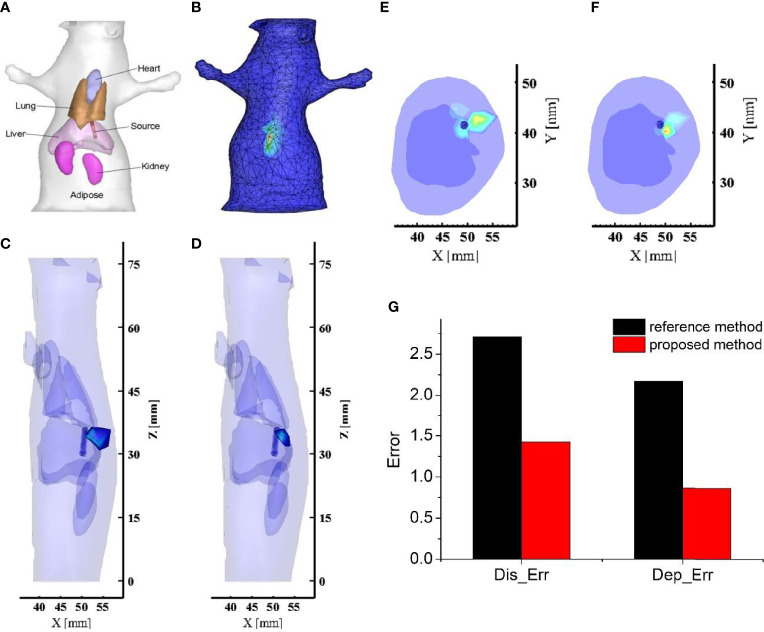
The reconstructed results and related quantitative analysis for the mouse based *in vivo* experiment. **(A)** Physical model of the mouse; **(B)** Light flux distribution on the mouse surface. **(C, D)** The 3D view of reconstructed images obtained by the reference method and the proposed method respectively; The long blue cylinder is the actual light source obtained by μCT, and the colored tetrahedrons are the reconstructed source; **(E, F)** The cross-sectional view of reconstructed images obtained by the reference method and the proposed method respectively. **(G)** The quantitative analysis of these reconstructed results. The black bars represent the quantitative indicators of the reference method, and the red bars are those of the proposed method.

**Figure 4A** shows the anatomical structure of the mouse with the organs highlighted, and these organs were used in the reconstruction. In order to reconstruct the internal light source, the collected single-view image by the CCD camera needs to map onto the mouse surface. Here, the Lambert source theory based energy mapping method was used (30), and the obtained light flux distribution on the mouse surface was shown in **Figure 4B**. In the reconstruction, the mouse model was discretized into a mesh consisting of 18,235 tetrahedrons and 3,731 nodes. Based on the light flux distribution on the mouse surface and the discretized nodal information, the internal artificial source was reconstructed by the reference and proposed methods respectively, with the results presented in **Figures 4C** **–F**. **Figures 4C** **, D** show a 3D view of the reconstructed results, which visualises the accurate positioning of the light source. **Figures 4E** **, F** give a cross-sectional view of the reconstructed result, such that the reconstructed information on the depth of the light source can be seen in this view. We found that the energy distribution of the light source reconstructed by the proposed method was more concentrated near the actual artificial source (**Figure 4D**), while the results obtained by the reference method had high energy regions far from the artificial source and close to the body surface towards the detection plane (**Figure 4C**). Furthermore, quantitative analysis in localization error and depth resolving error were plotted in **Figure 4G**. From the results presented in **Figure 4**, we can obtain almost the same conclusion as the numerical simulation. The reconstructed result obtained by the proposed method is greatly improved in both localization and depth resolving ability compared with the traditional DE-based method. In terms of the localization accuracy, the localization error was reduced by 1.9 times by using the proposed method. For the depth resolving ability, the proposed method can improve the depth resolving accuracy by 2.51 times. In particular, the proposed method reconstructed the center of the light source closer to the actual one and overcame the problem that the reconstruction results of the reference method tend to the surface of the object on the side of the detection plane. The reason for the good results was that the proposed method compensated for the loss of the signal emitted from the deep light source to the detection plane. In addition to the advantages in localization and depth resolving accuracy, we also found that the intersection volume between the reconstructed light source and the actual one was larger than that of the reference method. These results are completely consistent with the simulation results, and strongly proved the applicable potential of the proposed method in *in vivo* living animal imaging.

## Conclusion

In summary, a prior compensation algorithm was proposed for CLT reconstruction based on depth calibration strategy. This method is more suitable for data acquisition of commercial imaging system, in which a single-view image is often acquired for the reconstruction. Single-view image-based reconstruction will result in the loss of other views of signals. The farther away from the detection plane, the greater the loss of the luminescent signals can be. Thus, the proposed method designed a depth calibration matrix to calibrate the deviation of the depth reconstruction caused by the lack of information. With the help of this strategy, the inaccuracy of CLT reconstruction from single-view image can be improved. The validity and superiority of the prior compensation algorithm were verified using the numerical simulation with the light source at different depths. We also proved the potential of the proposed method in the *in vivo* applications with the artificial radioactive source based *in vivo* mouse experiment. There are some shortcomings for current algorithm. For example, in the proposed method, DE is used as the forward model for Cerenkov luminescence transmission, and DE has a limited range of applicability. The next step should incorporate higher order approximation equations or hybrid light propagation models as the forward model. For the construction of the compensation matrix, the center of the light source can be used as the exact starting point for compensation. However, how to determine the light source center also happens to be a problem that needs to be solved for CLT reconstruction, so the exact light source center location is unknown. In the following improvement, the determination of the starting point for compensation can be updated by integrating it into the iterative process of reconstruction. All in all, we believe that the proposed method will further promote the preclinical applications of CLT, especially in large-scale organisms. Our future prospective studies will focus on the biomedical applications of the prior compensation algorithm.

## Data Availability Statement

The original contributions presented in the study are included in the article/supplementary material. Further inquiries can be directed to the corresponding author.

## Ethics Statement

The animal study was reviewed and approved by Xi’an Jiaotong University Animal Care and Use Committee, China (No. XJTULAC2016-412).

## Author Contributions

LW designed the structure and prepared the manuscript. XH and JY modified the manuscript. All authors contributed to the article and approved the submitted version.

## Funding

This study was funded by the National Natural Science Foundation of China under Grants 61971350, 62101439, 61901374, 61906154, and 11871321, Natural Science Foundation of Shaanxi under Grants 2019JQ-724, Postdoctoral Innovative Talents Support Program under Grants BX20180254, Scientific and Technological projects of Xi’an under Grant 201805060ZD11CG44, and Education Department Served Local Special Projects under Grant 16JF026.

## Conflict of Interest

The authors declare that the research was conducted in the absence of any commercial or financial relationships that could be construed as a potential conflict of interest.

## Publisher’s Note

All claims expressed in this article are solely those of the authors and do not necessarily represent those of their affiliated organizations, or those of the publisher, the editors and the reviewers. Any product that may be evaluated in this article, or claim that may be made by its manufacturer, is not guaranteed or endorsed by the publisher.
